# Inability to contact opioid agonist therapy prescribers during the COVID-19 pandemic in a Canadian setting: a cross-sectional analysis among people on opioid agonist therapy

**DOI:** 10.1186/s13722-022-00354-x

**Published:** 2022-12-15

**Authors:** Soroush Moallef, Kora DeBeck, Nadia Fairbairn, Zishan Cui, Rupinder Brar, Dean Wilson, Cheyenne Johnson, M.-J. Milloy, Kanna Hayashi

**Affiliations:** 1 British Columbia Centre on Substance Use, St. Paul’s Hospital, 400-1045 Howe Street, Vancouver, BC V6Z 2A9 Canada; 2 School of Public Policy, Simon Fraser University, Burnaby, BC Canada; 3 Department of Medicine, University of British Columbia, Vancouver, BC Canada; 4 Vancouver Coastal Health Authority, Vancouver, BC Canada; 5 School of Nursing, University of British Columbia, Vancouver, BC Canada; 6 Faculty of Health Sciences, Simon Fraser University, Burnaby, BC Canada

**Keywords:** COVID-19, Opioid agonist therapy, Medication for opioid use disorder, Overdose

## Abstract

**Background:**

The COVID-19 pandemic and consequent public health response may have undermined key responses to the protracted drug poisoning crisis, including reduced access to opioid agonist therapy (OAT) among people with opioid use disorder. Our study objectives were to estimate the prevalence of and identify factors associated with inability to contact OAT prescribers when in need among people on OAT in a Canadian setting during the dual public health crises.

**Methods:**

Survey data were collected from three prospective cohort studies of community-recruited people who use drugs between July and November 2020, in Vancouver, Canada. A multivariable logistic regression analysis was used to identify potential factors associated with inability to contact OAT prescribers among patients who accessed OAT in the past 6 months.

**Results:**

Among 448 respondents who reported accessing OAT in the past 6 months, including 231 (54.9%) men, 85 (19.0%) reported having been unable to contact OAT prescribers when needed, whereas 268 (59.8%) reported being able to talk to their prescriber when needed, and 95 (21.2%) reported that they did not want to talk to their medication prescriber in the previous 6 months. Among those who reported inability to contact prescribers, 45 (53.6%) reported that their overall ability to contact prescribers decreased since the start of the pandemic. In multivariable analyses, factors independently associated with inability to talk to OAT prescribers included: chronic pain (Adjusted Odds Ratio [AOR] = 1.82; 95% Confidence Interval [CI] 1.02, 3.27), moderate to severe symptoms of depression or anxiety (AOR = 4.74; 95% CI 2.30, 9.76), inability to access health/social services (AOR = 2.66; 95% CI 1.41, 5.02), and inability to self-isolate or socially distance most or all of the time (AOR = 2.13; 95% CI 1.10, 4.14).

**Conclusions:**

Overall, approximately one fifth of the sample reported inability to contact their OAT prescribers when needed, and those people were more likely to have co-occurring vulnerabilities (i.e., co-morbidities, inability to access health/social services) and higher vulnerability to COVID-19. Interventions are needed to ensure optimal access to OAT and mitigate the deepening health inequities resulting from the COVID-19 pandemic and the escalating drug poisoning crisis.

## Background

People who use unregulated drugs (PWUD) are experiencing a deepening of health inequities resulting from the ongoing COVID-19 pandemic and the protracted drug poisoning crisis. Settings across Canada and the United States have documented unprecedented rates of fatal overdoses since the start of the pandemic [1–3], including a 58% increase in Canada and up to 60% increase in some jurisdictions in the US compared to the months prior to the pandemic [2]. Although the cause of the drug poisoning crisis is complex and multifactorial, the primary driver continues to be the contamination of the unregulated drug supply, most prominently with potent synthetic opioids such as fentanyl and its analogues [1, 3]. In addition, the unregulated drug supply has been increasingly contaminated in part owing to COVID-19 related border disruptions and drug shortages [4]. One of the primary interventions to address the poisoning crisis has been the expansion of opioid agonist therapy (OAT) to clinically manage opioid use disorder [5]. Indeed, a systematic review and meta-analysis has shown that retention in OAT is associated with significant reductions in risk of all-cause and overdose-related mortality [6]. However, OAT remains underutilized due to low retention rates [7–9], and access remains suboptimal due to a range of barriers, including: stigma and discrimination; onerous restrictions on treatment access including high out-of-pocket costs; daily witnessed ingestion at pharmacies or clinics; long wait times; and fragmented services (e.g., disconnected from primary care services) [10–15]. Qualitative interviews among providers of OAT in Canada have also noted that the biomedical focus of OAT and emphasis on abstinence hinders OAT treatment from being more patient- centred [11].

In an effort to minimize treatment disruptions and accommodate COVID-19 infection control mitigation strategies (i.e., physical distancing), jurisdictions across Canada and the US updated clinical practice guidelines for OAT (e.g., take-home doses, medication delivery) [16–18]. However, national qualitative studies in Canada during the first wave of the pandemic (May–July 2020), have reported that disruptions to OAT and other health and social services (e.g., harm reduction sites, access to inpatient treatment) were commonplace and largely attributed to public health infection control strategies such as closure of non-essential services, stay-at-home orders, and physical distancing) [19, 20], Of concern, PWUD have reported treatment gaps in OAT that rendered individuals vulnerable to overdose, among other preventable negative physical and mental health outcomes [20]. Building on these previous qualitative studies, the purpose of this quantitative survey study was to assess access to OAT prescribers among OAT patients during the dual public health crisis of COVID-19 and drug poisoning, and amid British Columbia’s provincial expansion of prescription guidelines. Specifically, our study objective was to identify factors associated with inability to contact OAT prescribers when in need among OAT patients derived from a community-recruited sample of PWUD in Vancouver, Canada, a setting where oral OAT (e.g., buprenorphine/naloxone and methadone) are widely available through primary care clinics and family physicians’ offices and dispensed by community pharmacies.

## Methods

### Study sample

Data for this study were drawn from three ongoing prospective cohort studies of PWUD in Vancouver: the Vancouver Injection Drug Users Study (VIDUS), the AIDS Care Cohort to evaluate Exposure to Survival Services (ACCESS) and the At-Risk Youth Study (ARYS). These long-running (since 2005) and well-characterized community-recruited cohorts have been described elsewhere [21–23]. In brief, VIDUS enrols HIV-seronegative adults (≥ 18 years of age) who injected drugs in the month before enrolment. ACCESS enrols HIV-seropositive adults who used an unregulated drug other than or in addition to cannabis in the month prior to enrolment. ARYS enrols street-involved youth aged 14–26 who used an unregulated drug other than or in addition to cannabis in the month prior to enrolment. All cohorts recruit participants through street outreach and word-of-mouth. The studies use harmonized data collection and follow-up procedures to allow for merged data analyses. All three cohorts administer questionnaires by trained interviewers at equal follow-up frequency (i.e., every 6 months). At each study visit, participants receive a CAD $40 honorarium. Between July and November 2020, due to the COVID-19 pandemic, all study follow-up interviews were conducted remotely over the phone. The present analysis drew data from these phone interviews and restricted an analytic sample to those who reported having accessed OAT in the past 6 months. All three cohorts have received ethics approval by University of British Columbia/Province Health Care Research Ethics Board.

### Study measures

The primary outcome of interest was self-reported ability to contact OAT prescriber when in need in the past 6 months, derived from a survey question: “In the last 6 months, was there a time when you wanted to talk to your medication prescriber but were not able?” Responses included: “unable to talk”, “able to talk”, “did not want to talk”. For regression analyses, our primary comparison was “unable to talk” vs. “able to talk”, while we also compared “did not want to talk” vs. “able to talk” as secondary analyses.

The explanatory variables of interest included the following socio-demographic variables: age (per year older, continuous); ethnicity/ancestry (white vs. IBPOC [Indigenous, Black, and People of Colour]); self-identified gender (male vs. female and non-binary gender); education (< secondary school vs. ≥ secondary school); residence in the Downtown Eastside neighbourhood of Vancouver (DTES), an area with high concentration of unregulated drug use and related services (e.g., harm reduction and low-barrier OAT services); and homelessness. Drug use related variables included: injection drug use; ≥ daily use of unregulated opioids (defined as fentanyl, “down” [the local term for unregulated opioids] or heroin [injection/non-injection] use); ≥ daily non-medical use of prescription opioids; ≥ daily use of stimulants, defined as powder/crack cocaine or crystal methamphetamine use; ≥ daily use of cannabis; and non-fatal overdose. Other health-related variables included: anxiety/depression symptoms assessed using the Patient-Reported Outcomes Measurement Information System (PROMIS) short form (moderate/severe vs. mild/none) [24]; and chronic pain, defined as persistent or recurrent pain lasting longer than 3 months, a definition that is consistent with the International Association for the Study of Pain [25]. Other social/structural exposures of interest included: incarceration, police confrontations (defined as stopped, searched, or detained by the police), and experience of physical or sexual violence. We also included variables related to experiences that occurred since the start of the COVID-19 pandemic, including: increase in violence or sexual assault, increase in difficulty accessing health or social services (e.g., housing or counselling), and inability to self-isolate or social distance (all the time/most of the time vs. some of the time/not at all). Except for age, gender, education, and ethnicity/ancestry, all variables referred to the previous 6 months and coded as yes vs. no unless otherwise indicated.

### Statistical analysis

As a first step, we tested for differences in the descriptive characteristics of the sample across the three categories in the outcome variable using the Pearson’s χ^2^ test and Fisher’s test for counts < 5 (for categorical variables) or Kruskal Wallis test (for continuous variables) as appropriate. Missing observations were minimal and excluded from the denominator for calculations involving percentages. Bivariable logistic regression was used to identify the crude relationships between factors associated with inability to contact OAT prescribers. The multivariable model was fit using an a priori-defined statistical protocol based on examining the AIC and type III p-values. First, a preliminary model was constructed using all variables significantly associated with the outcome in bivariable analyses at p < 0.10. Next, each variable with the highest p-value was removed sequentially, with the final model including the set of variables associated with the lowest AIC score. We also examined changes in the ability to contact OAT prescribers since the start of the COVID-19 pandemic stratified by our primary outcome variable using the following question: “Has your ability to talk to your medication prescriber changed since the beginning of the COVID public health emergency?” Responses included: “Yes, access/ability has increased”, “Yes, access/ability has decreased”, “No”.

We also conducted two exploratory analyses to aid the interpretation of the results of the primary analyses. In order to help clarify whether the inability to contact OAT prescribers resulted from prescriber- or patient-related factors, we conducted an exploratory analysis to compare participants’ reports of whether any clinic visits or service for OAT were cancelled due to the COVID-19 pandemic in the past month across the three categories of our primary outcome variable. Lastly, in the second exploratory analysis, among those who received methadone as their most recent OAT medication (n = 316), we examined perceived levels of the medication dosage (about right, too low, or too high) stratified by our primary outcome variable. All p-values were two-sided and all statistical analyses were conducted using SAS version 9.4 (SAS Institute, Cary, North Carolina, United States).

## Results

In total, 884 participants completed an interview between July and November 2020, of whom 448 (50.7%) participants reported being on OAT in the past 6 months and thus formed the analytic sample. The analytic sample was comprised of 246 (54.9%) self-identified males, 273 (61.4%) self-identified as white, 158 (35.5%) self-identified as Indigenous and 14 (3.1%) self-identified as POC. The sample was further comprised of 227 VIDUS (50.7%), 140 (31.3%), ACCESS and 81 (18.1%) ARYS participants and the median age was 49 (1st and 3rd quartile: 37, 57) years. Overall, 235 (52.0%) resided in the DTES neighbourhood in the past 6 months, 151 (33.4%) in other neighbourhoods within the city of Vancouver, and 66 (14.6%) outside of the city of Vancouver.

As shown in Table 1, participants reported accessing a range of OAT, with Methadone (70.5%) being the most prescribed medication. Overall, 85 (19.0%) reported inability to talk to their medication prescriber when needed in the past 6 months when needed, whereas 268 (59.8%) reported being able to talk to their medication prescriber when needed, and 95 (21.2%) reported that they did not want to talk to their medication prescriber in the past 6 months. In addition, 61 (13.8%) reported that their ability to contact their medication prescriber had decreased since the start of the COVID-19 pandemic.

**Table 1:** Sample characteristics stratified by inability to contact opioid agonist treatment (OAT) prescriber when needed among 448 people who accessed OAT in the past 6 months in Vancouver, Canada, between July and November 2020

Characteristic	Total	Inability to contact OAT prescriber (%)			p-value
		Unable to Talk	Able to talk	Did not want to talk	
	448 (100%)	85 (19.0%)	268 (59.8%)	95 (21.2%)	
Most recent OAT prescribed					
Methadone	316 (70.5)	59 (69.4)	184 (68.7)	73 (76.8)	–
Buprenorphine/naloxone (suboxone)	34 (7.6)	8 (9.4)	23 (8.6)	3 (3.16)	–
Long-acting buprenorphine	1 (0.2)	0	1 (0.4)	0	–
Injectable OAT	29 (6.5)	3 (3.5)	20 (7.5)	6 (6.3)	–
SROM	37 (8.3)	6 (7.1)	28 (10.5)	3 (3.1)	–
Cancellation of visits or service for OAT due to the COVID-19 pandemic^a^	43 (9.6)	17 (20.0)	15 (5.6)	11 (11.6)	< 0.001
Age (median, Q1–Q3)	49 (37–57)	40 (31–53)	50 (39–58)	49 (37–57)	0.001
Male (vs. female and non-binary)	246 (54.9)	45 (52.9)	139 (51.9)	62 (65.3)	0.073
White (vs. IBPOC*)*	273 (61.4)	65 (76.5)	157 (59.0)	51 (54.3)	0.008
> Secondary school	209 (47.4)	39 (46.4)	130 (49.1)	40 (43.5)	0.685
Chronic pain^b^^,c^	194 (43.3)	48 (56.5)	113 (42.3)	33 (34.7)	0.011
Moderate/severe depression or anxiety^d^	220 (52.6)	62 (81.6)	116 (46.0)	42 (46.7)	< 0.000
DTES residence^b^	235 (52.5)	41 (48.2)	145 (54.1)	49 (51.6)	0.629
Homeless^b^	55 (12.3)	13 (15.5)	29 (10.9)	13 (13.7)	0.481
Incarceration^b^	10 (2.2)	2 (2.4)	5 (1.9)	3 (3.2)	0.765
Negative police encounter^b,e^	17 (3.8)	5 (6.0)	8 (3.0)	4 (4.2)	0.459
Non-fatal overdose^b^	68 (15.3)	20 (24.1)	31 (11.6)	17 (17.9)	0.016
Self-reported substance use^b^					
Daily unregulated opioids use^f^	203 (45.4)	44 (51.8)	113 (42.3)	46 (48.4)	0.252
Daily prescription opioids use^g^	13 (2.9)	4 (4.7)	4 (1.5)	5 (5.3)	0.096
Daily stimulant use	150 (33.6)	35 (41.2)	81 (30.3)	34 (35.8)	0.160
Injection drug use	264 (58.9)	54 (63.5)	152 (56.7)	58 (61.1)	0.481
Experiences since beginning of COVID-19					
Increase in violence	35 (8.1)	12 (14.5)	19 (7.4)	4 (4.4)	0.043
Increase in difficulty accessing health/social services	88 (20.3)	38 (45.8)	39 (15.2)	11 (11.8)	< 0.000
Inability to self-isolate or social distance	106 (23.8)	29 (34.5)	46 (17.2)	31 (32.6)	< 0.000
Perceived levels of medication doses among those on methadone (n = 316)					
About right	218 (73.9)	32 (58.2)	140 (81.4)	46 (67.6)	0.004
Too low	62 (21.0)	18 (32.7)	28 (16.3)	16 (23.5)	
Too high	15 (5.1)	5 (9.1)	4 (2.3)	6 (8.8)	

Missing observations were minimal and excluded from the denominator when calculating percentages

*DTES* downtown eastside, *PO* prescription opioid, *SROM* slow-release oral morphine, *IBPOC* indigenous, black, and people of colour

^a^Denotes behaviors/events in the past month

^b^Denotes behaviors/events in the past 6 months

^c^Chronic Pain is defined using the international pain guidelines (persistent or recurrent pain lasting longer than 3 months)

^d^Patient-Reported Outcomes Measurement Information System (PROMIS) short form was used to assess anxiety/depression (moderate/severe vs. mild/none)

^e^Negative police encounter refers to being stopped, searched, or detained by the police

^f^Daily unregulated opioid use includes the use of fentanyl, down unspecified or heroin (injection/non-injection)

^g^Daily stimulant use included the use of daily crack, daily methamphetamine*,* and daily cocaine in the last 6 months

In the first multivariable model (Table 2), factors significantly associated with inability to talk to medication prescribers when needed (compared to ability to talk) included: younger age (Adjusted Odds Ratio [AOR]; 0.94; 95% Confidence Interval [CI] 0.91–0.97), white ethnicity/ancestry (AOR: 3.35; 95% CI 1.72–6.51), chronic pain (AOR = 1.82; 95% CI 1.02–3.27); moderate/severe symptoms of depression or anxiety (AOR = 4.66; 95% CI 2.27–9.57); inability to self-isolate or socially distance all or most of the time (AOR: 2.13; 95% CI 1.10–4.14); and increased inability to access health/social services during the COVID-19 pandemic (AOR: 2.66; 95% CI 1.41–5.02). In the second multivariable model (Table 2), only inability to self-isolate or socially distance all or most of the time (OR: 2.21; 95% CI 1.24–3.94) was retained in the model selection procedure and was positively associated with not wanting to talk to prescribers (compared to ability to talk).

**Table 2:** Univariable and multivariable logistic regression analyses of factors associated with ability to contact Opioid Agonist Treatment (OAT) prescriber when needed among 448 people who accessed OAT in the past 6 months in Vancouver, Canada, between July 2020 and November 2020

Variable	Model 1		Model 2	
	Unable to talk vs. Able to talk		Did not want to talk vs. Able to talk	
	OR (95% CI)	AOR (95% CI)	OR (95% CI)	AOR (95% CI)
Age (per year increase)	0.96 (0.94, 0.98)	0.94 (0.91, 0.97)	0.99 (0.97, 1.01)	–
Male (vs. female and non-binary)	1.05 (0.63, 1.74)	–	1.74 (1.07, 2.83)	–
White (vs. IBPOC)	2.26 (1.29, 3.94)	3.35 (1.72, 6.51)	0.82 (0.51, 1.32)	–
≥ Secondary school	0.90 (0.55, 1.47)	–	0.80 (0.50, 1.29)	–
Chronic pain^a,b^	1.77 (1.08, 2.89)	1.82 (1.02, 3.27)	1.77 (1.08, 2.89)	–
Moderate/severe depression or anxiety^c^	5.19 (2.76, 9.76)	4.66 (2.27, 9.57)	5.19 (2.76, 9.76)	–
DTES residence^a^	0.79 (0.48, 1.29)	–	0.79 (0.48, 1.29)	–
Homeless^a^	1.50 (0.74, 3.04)	–	1.50 (0.74, 3.04)	–
Incarceration^a^	1.28 (0.24, 6.71)	–	1.28 (0.24, 6.71)	–
Negative police encounter^a,d^	2.04 (0.65, 6.42)	–	2.04 (0.65, 6.42)	–
Non-fatal overdose^a^	2.42 (1.29, 4.52)	–	2.42 (1.29, 4.52)	–
Self-reported substance use^a^				
Daily unregulated opioids use^e^	1.46 (0.90, 2.39)	–	1.46 (0.90, 2.39)	–
Daily PO use	3.23 (0.79, 13.22)	–	3.23 (0.79, 13.22)	–
Daily stimulants use^f^	1.61 (0.97, 2.66)	–	1.61 (0.97, 2.66)	–
Injection drug use^g^	1.33 (0.80, 2.20)	–	1.33 (0.80, 2.20)	–
Experiences since beginning COVID-19				
Increase in violence	2.11 (0.98, 4.55)	–	2.11 (0.98, 4.55)	–
Increase in difficulty accessing health/social services	4.72 (2.72, 8.18)	2.66 (1.41, 5.02)	4.72 (2.72, 8.18)	–
Inability to self-isolate or social distance	2.53 (1.46, 4.39)	2.13 (1.10, 4.14)	2.53 (1.46, 4.39)	2.21 (1.24, 3.94)

*AOR* adjusted odds ratio, *CI* confidence interval, *IBPOC* indigenous, black, and people of colour, *DTES* downtown eastside, *OAT* opioid agonist treatment, *OR* odds ratio, *PO* prescription opioid

^a^Denotes behaviors/events in the past 6 months

^b^Chronic Pain is defined using the international pain guidelines (persistent or recurrent pain lasting longer than 3 months)

^c^Patient-Reported Outcomes Measurement Information System (PROMIS) short form was used to assess anxiety/depression (moderate/severe vs. mild/none)

^d^Negative police encounter refers to being stopped, searched, or detained by the police

^e^Daily unregulated opioid use includes the use of fentanyl, down unspecified or heroin (injection/non-injection)

^f^Daily stimulant use included the use of daily crack, daily *methamphetamine,* and daily cocaine in the last 6 months

^g^Injection Drug Use includes those who injected drugs at least once in the last 6 months

**Fig. 1:**
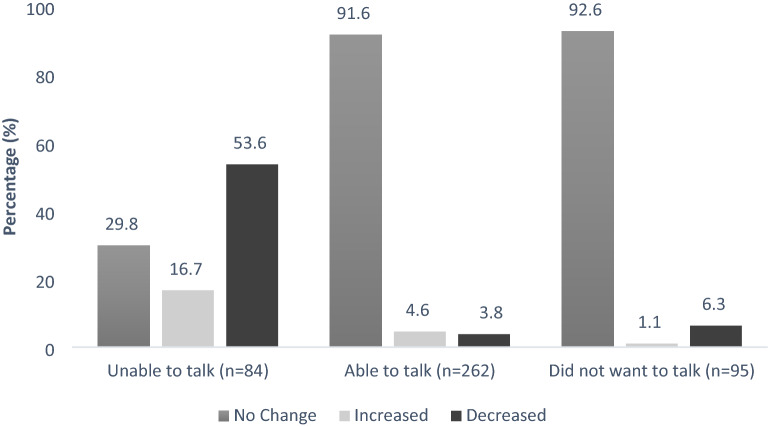
Changes in the ability to talk to medication prescriber since the beginning of COVID-19 pandemic amongst respondents who were unable to talk, able to talk, and did not want to talk to their medication prescriber (n = 441)

In the sub-analysis, among those who reported inability to talk to prescribers, the majority (53.6%, n = 45) reported that their access to prescribers had decreased since the start of the pandemic, while the majority of those who reported ability to talk to prescribers (91.6%, n = 240) and the majority of those who did not want to talk to prescribers (92.6%, n = 88) reported that their access to prescribers had remained unchanged since the start of the pandemic (Fig. 1). Further, in the exploratory analysis, as shown in Table 1, 20% of those who reported inability to contact prescribers reported that their OAT clinic visits or service were cancelled due to the COVID-19 pandemic in the past month, while 5.6% and 11.6% of those who reported ability to contact their prescribers and who did not want to talk to prescribers, respectively did so (p < 0.001). Among a sub-sample of those who received methadone (n = 316), 32.7% those who reported inability to contact prescribers perceived their methadone doses as too low, whereas 16.3% and 23.5% of those who were able to talk and who did not want to talk to prescribers, respectively did so (p = 0.004).

## Discussion

Between July and November 2020, approximately one in five people on OAT in our community-recruited sample of PWUD reported inability to contact their OAT providers when needed in the past 6 months. Our multivariable findings showed that those who were unable to talk to their OAT prescribers were more likely to have co-morbidities (such as chronic pain and mental health issues), perceived greater health/social services disruptions since the onset of the COVID-19 pandemic, and were unable to ensure COVID-19 precautions. In addition, about half (53%) of those who reported inability to contact their prescriber perceived that their overall access to providers had decreased since the start of the pandemic.

The findings that about a fifth of our sample reported difficulty contacting their prescriber when needed and that over half of these individuals perceived their overall access to prescribers as reduced since the onset of the pandemic is concerning. Because our study did not examine specific reasons for the inability to contact prescribers, we are unable to disentangle provider/clinic- (e.g., service cancellations) and patient-related factors (e.g., social-structural factors shaping their living environment) related to the inability. Both these types of factors likely co-existed, and in some cases, they were intertwined during the COVID-19 pandemic. Our exploratory analysis shows that those who were unable to contact their prescribers when needed were disproportionately affected by cancellation of OAT clinic visits or service in the past month compared to those who were able to talk or did not want to talk to prescribers (20% vs. 5.6% vs. 11.6%, respectively; p < 0.001). This result indicates that provider-level factors played a role in some participant’s inability to contact prescribers but not all. An inability to contact prescribers when needed could indicate or result in suboptimal treatment experience, which, if left unaddressed, could be a precursor to disengagement with OAT services and lead to more use of unregulated and contaminated drugs. For example, our exploratory results focusing on those receiving methadone demonstrated that one aspect of suboptimal treatment experience, that is perceiving that medication dosage is insufficient, was the highest among those who were unable to contact their methadone prescribers (32.7%) compared to those who were able (16.3%) or did not want to talk to their prescribers (23.5%; p = 0.004). Given that lower methadone dosages have been shown to be a strong predictor of treatment discontinuation [26–30], and also because the concentration of fentanyl in the unregulated supply has increased during the COVID-19 pandemic [3], it is particularly important to address any patient-provider communication gaps and the insufficient medication dosages perceived by patients to help reduce patients’ exposure to unregulated opioids with high concentrations of fentanyl.

Our multivariable analysis found that the perceived communication challenge with OAT prescribers were concentrated among people who reported co-morbidities including chronic pain and moderate/severe depression or anxiety. Of concern, chronic pain and mental health issues are persistent unmet healthcare needs among PWUD [12, 15, 31–33], and consistently linked to higher levels of self-medication, greater drug use severity, and engagement with the unregulated and contaminated drug supply. Research has also linked greater pain severity and untreated psychological disorders with discontinuation of OAT or inability to access treatment [32, 34]. Our findings strengthen the need for improved management of both mental health and chronic pain issues among those on OAT. To this end, many jurisdictions have yet to integrate OAT services into primary care and mental health services, which remains a persistent structural barrier for providers to provide and patients to receive comprehensive and optimal treatment for co-occurring health issues, particularly chronic pain and mental health among PWUD [12, 15].

Our findings also showed that those who reported an increased difficulty in accessing health/social services in general (e.g., counselling or housing) and those who were unable to self-isolate or social distance all or most of the time were more likely to report inability to contact OAT providers. These findings indicate that those who experienced difficulty contacting their OAT prescribers may also be at increased risk of not only overdose (due to suboptimal OAT) but also COVID-19 infection (due to being unable to self-isolate or social distance) and other negative health outcomes (due to difficulty accessing health/social services in general).

Overall, the findings in this study underscore the need to mitigate treatment gaps and subsequent overdose risk as a potential result of suboptimal treatment experience. Inability to contact OAT prescribers when needed undermines the intended goal of the expanded OAT prescription guidelines in this setting and interventions are needed to improve communication between OAT patients and prescribers. Research suggests that multi-level interventions are needed to remedy the existing barriers to accessing OAT [10–15], as well as new challenges posed by the pandemic [19, 20], which should include but not limited to: routine risk assessment of OAT provisions, access to telemedicine (including provisions of technology) [11], and expansion of mobile OAT options. It is worth noting that our primary outcome measure, inability to contact OAT prescribers, is not an objective measure of service access (e.g., whether they accessed OAT or not) but is assessing one aspect of patient-reported satisfaction with OAT in relation to patient-provider communication. Broadly speaking, a key goal of all national health systems according to the World Health Organization is to respond to the legitimate expectations of individuals (otherwise known as health system responsiveness), which includes prompt attention to individual care needs [35, 36]. In this regard, patient-reported (in)ability to communicate with OAT prescribers when needed can be conceptualized as an important element shaping the individual’s treatment experiences and health system responsiveness. Overall, the findings in this study underscore the need to address patient-provider communication gap and improve suboptimal treatment experiences, which could lead to negative health outcomes.

Our findings should be considered with several limitations. First, the non-random nature of our sample reduces the ability to generalize our results to all PWUD in our study setting. Study data were also derived from telephone-based interviews, which may have underestimated the level of inability to contact providers, as those without a known telephone number could not be reached and may be at greater risk for having inability to access providers. In addition, all data were self-reported, and therefore might be influenced by reporting bias. We are also unable to discern potential reasons for why some participants reported an inability to talk to prescribers and not wanting to talk to prescribers. We are also unable to discern potential reasons for why some participants reported not wanting to talk to prescribers, an important area for future research as it could be an indicator of sub-optimal treatment. Additionally, there were shifts towards virtual and remote delivery of services that accompanied the COVID-19 pandemic in our setting, which we are unable to account for in our findings. If participants did access telemedicine services, we would expect a potential improved ability to contact service providers when needed, however future research is needed to account for the impact of telemedicine and its utility in improving communication between patients and providers. Lastly, our analytic sample was restricted to those who reported having accessed OAT in the past 6 months, which may have included experiences that occurred prior to COVID-19 for those with interview dates in July/August 2020 (n = 117).

## Conclusion

Our findings show that among OAT patients derived from a community-recruited sample of PWUD in Vancouver, Canada, patient’s ability to contact OAT prescribers was negatively impacted during the first 6 months of the COVID-19 pandemic. Overall, one in five OAT patients reported inability to contact prescribers when needed in the past 6 months, of which over half reported that their ability to reach their prescribers had decreased since the start of the pandemic. Individuals who reported inability to talk to prescribers were characterized by co-occurring vulnerabilities, including health issues (e.g., chronic pain, mental health), greater perceived barriers to accessing health/social services during the pandemic, and inability to self-isolate or social distance all or most of the time. Expanded efforts are needed to address the patient-provider communication gap and the resultant suboptimal treatment experiences.

## Data Availability

The datasets used and/or analysed during the current study are available from the corresponding author on reasonable request and with permission of the University of British Columbia/Providence Health Care Research Ethics Board.
